# Expression of cholecystokinin by neurons in mouse spinal dorsal horn

**DOI:** 10.1002/cne.24657

**Published:** 2019-02-20

**Authors:** Maria Gutierrez‐Mecinas, Andrew M. Bell, Fraser Shepherd, Erika Polgár, Masahiko Watanabe, Takahiro Furuta, Andrew J. Todd

**Affiliations:** ^1^ Institute of Neuroscience and Psychology, College of Medical, Veterinary & Life Sciences, University of Glasgow Glasgow United Kingdom; ^2^ Department of Anatomy Hokkaido University School of Medicine Sapporo Japan; ^3^ Department of Oral Anatomy and Neurobiology, Graduate School of Dentistry Osaka University Osaka Japan

**Keywords:** excitatory interneuron, gastrin‐releasing peptide, neurokinin B, neurotensin, RRID:AB_2298772, RRID:AB_2314928, RRID:AB_2533990, RRID:AB_2571674, RRID:AB_2571826, RRID:AB_2619988, RRID:AB_300798, substance P, thyrotropin‐releasing hormone

## Abstract

Excitatory interneurons account for the majority of dorsal horn neurons, and are required for perception of normal and pathological pain. We have identified largely non‐overlapping populations in laminae I‐III, based on expression of substance P, gastrin‐releasing peptide, neurokinin B, and neurotensin. Cholecystokinin (CCK) is expressed by many dorsal horn neurons, particularly in the deeper laminae. Here, we have used immunocytochemistry and in situ hybridization to characterize the CCK cells. We show that they account for ~7% of excitatory neurons in laminae I‐II, but between a third and a quarter of those in lamina III. They are largely separate from the neurokinin B, neurotensin, and gastrin‐releasing peptide populations, but show limited overlap with the substance P cells. Laminae II‐III neurons with protein kinase Cγ (PKCγ) have been implicated in mechanical allodynia following nerve injury, and we found that around 50% of CCK cells were PKCγ‐immunoreactive. Neurotensin is also expressed by PKCγ cells, and among neurons with moderate to high levels of PKCγ, ~85% expressed CCK or neurotensin. A recent transcriptomic study identified mRNA for thyrotropin‐releasing hormone in a specific subpopulation of CCK neurons, and we show that these account for half of the CCK/PKCγ cells. These findings indicate that the CCK cells are distinct from other excitatory interneuron populations that we have defined. They also show that PKCγ cells can be assigned to different classes based on neuropeptide expression, and it will be important to determine the differential contribution of these classes to neuropathic allodynia.

## 1 INTRODUCTION

The spinal dorsal horn contains numerous interneurons, which are involved in processing somatosensory information (Abraira & Ginty, 2013; Braz, Solorzano, Wang, & Basbaum, 2014; Peirs & Seal, 2016; Todd, 2010). These can be divided into two broad classes: excitatory and inhibitory cells (Todd, 2010, 2017). In the mouse, excitatory (glutamatergic) interneurons account for ~75% of the neurons in laminae I‐II and 60% of those in lamina III (Polgár, Durrieux, Hughes, & Todd, 2013). Although the roles of these cells are not fully understood, it has been shown those in laminae I‐II are required for acute mechanical pain and development of mechanical allodynia (Christensen et al., 2016; Duan et al., 2014; Wang et al., 2013).

Dorsal horn excitatory interneurons are functionally diverse, and there have been many attempts to define distinct populations among these cells (Todd, 2017). Most recent studies have used neurochemical criteria, in particular the expression of neuropeptides. We have identified four largely non‐overlapping populations in laminae I‐II, based on the expression of neurotensin, neurokinin B (NKB), substance, P and gastrin‐releasing peptide (GRP), and have shown that between them, they account for over half of the excitatory interneurons in this region (Gutierrez‐Mecinas et al., 2017; Gutierrez‐Mecinas, Furuta, Watanabe, & Todd, 2016). While the neurotensin and NKB cells can be identified by immunocytochemistry, we revealed the substance P cells by spinal injection of adeno‐associated viruses (AAVs) coding for Cre‐dependent expression cassettes into mice in which Cre recombinase was knocked into Tac1 (the gene for substance P). The GRP cells were identified by the presence of enhanced green fluorescent protein (eGFP) in a BAC transgenic mouse line (GRP::eGFP) in which eGFP is expressed under control of the GRP promoter (Gutierrez‐Mecinas, Watanabe, & Todd, 2014; Mishra & Hoon, 2013; Solorzano et al., 2015). Neurotensin and NKB neurons, which are concentrated in inner lamina II (lamina IIi) and lamina III, partly correspond to cells that express protein kinase Cγ (PKCγ; Gutierrez‐Mecinas et al., 2016). PKCγ‐expressing cells are of particular interest, because they are thought to form part of a circuit that is responsible for mechanical allodynia (Lu et al., 2013; Malmberg, Chen, Tonegawa, & Basbaum, 1997; Miraucourt, Dallel, & Voisin, 2007).

A recent transcriptomic study by Häring et al. (2018) identified 15 classes of excitatory dorsal horn neuron, and their results are broadly consistent with the findings described above, as their classes include cells with high levels of the mRNAs for neurotensin, NKB, and substance P. Another peptide that featured in their classification scheme was cholecystokinin (CCK), which was expressed in three separate populations of excitatory neurons. Early reports had indicated that neurons with CCK mRNA were most numerous in lamina III and scattered through the other laminae (Abelson & Micevych, 1991; Cortes, Arvidsson, Schalling, Ceccatelli, & Hokfelt, 1990; Schiffmann, Teugels, Halleux, Menu, & Vanderhaeghen, 1991), while a recent study found that CCK defined a specific population of excitatory neurons in the deeper laminae of the dorsal horn (Abraira et al., 2017).

The first aim of the present study was to estimate the proportion of excitatory neurons in laminae I‐III that expressed CCK. This was achieved with two approaches: immunocytochemistry with an antibody against the precursor pro‐CCK (Booker et al., 2017) and multiple‐labeling fluorescent in situ hybridization. We also examined the extent of overlap with the four populations of excitatory interneurons that we had identified in the superficial dorsal horn. Our preliminary studies indicated that some pro‐CCK‐positive neurons in laminae II‐III showed strong PKCγ‐immunoreactivity, and we therefore compared expression of neurotensin, CCK, and PKCγ, because neurotensin is also expressed by cells with high levels of PKCγ in this region (Gutierrez‐Mecinas et al., 2016). Finally, because Häring et al. (2018) reported that one of the CCK populations could be identified based on expression of thyrotropin‐releasing hormone (TRH), we looked for cells that contained mRNAs for both CCK and TRH and tested whether these corresponded to PKCγ‐expressing neurons.

## 2 MATERIALS AND METHODS

### 2.1 Animals

All experiments were approved by the Ethical Review Process Applications Panel of the University of Glasgow, and were performed in accordance with the UK Animals (Scientific Procedures) Act 1986.

Five wild‐type C57BL/6 mice (either sex, 18–27 g) and 3 GRP::eGFP mice (Gutierrez‐Mecinas et al., 2014; either sex, 19–27 g) were deeply anesthetized with pentobarbitone (20 mg i.p.) and perfused through the left cardiac ventricle with a fixative consisting of 4% freshly depolymerized formaldehyde in phosphate buffer. Mid‐lumbar spinal cord segments (L3–L5) were removed and postfixed for 2 hr at 4 °C in the same fixative before being processed for immunocytochemistry. Spinal cord tissue was also obtained from three male Tac1^Cre^ mice (19–22 g; Harris et al., 2014) that had received intraspinal injections of AAV (serotype 1) coding for Cre‐dependent eGFP (AAV.flex.eGFP; Penn Vector Core, Philadelphia, PA) into the lumbar enlargement, and had been used in a previous study (Gutierrez‐Mecinas et al., 2019). These mice had survived 8 days after the spinal injection before being perfused with the same fixative. Transverse sections from segments that included the injection sites were processed for immunocytochemistry.

Three wild‐type C57BL/6 mice (either sex, 18–20 g) were used for in situ hybridization experiments. The mice were decapitated under deep isoflurane anesthesia and spinal cords were removed and rapidly frozen on dry ice.

### 2.2 Immunocytochemistry, confocal microscopy, and analysis

Transverse spinal cord sections 60 μm thick were cut from the perfusion‐fixed tissue with a vibrating blade microtome and immersed in 50% ethanol for 30 min to enhance antibody penetration. These were then reacted for multiple‐labeling immunofluorescence staining as described previously (Gutierrez‐Mecinas et al., 2016). The primary antibodies used in this part of the study are listed in Table 1. Unless stated otherwise, sections were incubated for 3 days at 4 °C in primary antibodies diluted in PBS that contained 0.3 M NaCl, 0.3% Triton X‐100 and 5% normal donkey serum, and then overnight in appropriate species‐specific secondary antibodies (Jackson Immunoresearch, West Grove, PA) that were raised in donkey and conjugated to Alexa 488, Alexa 647, Rhodamine Red, Pacific Blue or biotin. All secondary antibodies were used at 1:500 (in the same diluent), apart from those conjugated to Rhodamine Red or Pacific Blue, which were diluted to 1:100 and 1:200, respectively. Biotinylated secondary antibodies were detected with either Pacific Blue conjugated to avidin (1:1,000; Life Technologies, Paisley, UK) or with a tyramide signal amplification (TSA) method (TSA kit tetramethylrhodamine NEL702001, PerkinElmer Life Sciences, Boston, MA). The TSA reaction was used to detect the NKB precursor preprotachykinin B (PPTB), as this method can be used to reveal cell bodies of NKB‐expressing dorsal horn neurons (Polgár, Furuta, Kaneko, & Todd, 2006). Following the immunocytochemical reaction, sections were mounted in antifade medium and stored at −20 °C.

**Table 1:** Antibodies used in this study

Antibody	Host and type	Antigen	Supplier/reference	Catalogue number	RRID	Dilution
Pro‐CCK	Rabbit polyclonal	Amino acids 107–115 of mouse pro‐CCK	Booker et al. (2017)		RRID:AB_2571674	1,000
NeuN	Mouse monoclonal	Purified cell nuclei from mouse brain	Millipore	MAB377	RRID:AB_2298772	1:500
NeuN	Guinea pig polyclonal	Amino acids 1–97 of mouse Fox‐3	Synaptic Systems	266004	RRID:AB_2619988	1:1,000
Neurotensin	Rat polyclonal	Synthetic neurotensin	Porteous et al. (2011)		RRID:AB_2314928	1:1,000
PKCγ	Guinea pig polyclonal	Amino acids 684–697 of mouse PKCγ	Yoshida et al. (2006)		RRID:AB_2571826	1:1,000
PPTB	Guinea pig polyclonal	Amino acids 95–116 of rat PPTB	Kaneko, Murashima, Lee, and Mizuno (1998)			1:5,000
Pax2	Rabbit polyclonal	Amino acids 188–385 of mouse Pax2	Life Technologies	716000	RRID:AB_2533990	1:500
eGFP	Chicken	Recombinant full‐length eGFP	Abcam	Ab13970	RRID:AB_300798	1:1,000

Sections from the GRP::eGFP mice were reacted with primary antibodies against eGFP, pro‐CCK, and NeuN (mouse antibody), and following incubation in secondary antibodies, they were counterstained with 4′6‐diamidino‐2‐phenylindole (DAPI) to label cell nuclei. Sections from the wild‐type mice were reacted with three different combinations of primary antibodies: (a) pro‐CCK, neurotensin, PKCγ, and NeuN (mouse antibody); (b) pro‐CCK, PPTB, NeuN (mouse antibody); (c) pro‐CCK, Pax2, NeuN (mouse antibody). For the third reaction, a sequential staining protocol was used, because both the pro‐CCK and Pax2 antibodies are raised in rabbit. In this case, sections were initially incubated in pro‐CCK and NeuN antibodies, which were revealed with fluorescent secondary antibodies conjugated to Alexa 488 and Alexa 647, respectively. They were then treated with unconjugated Fab' donkey anti‐rabbit IgG fragment for 6 hr to saturate binding sites for rabbit IgG, before being incubated for 2 days in the Pax2 antibody, which was subsequently revealed with secondary antibody conjugated to Rhodamine Red. Tissue from the Tac1^Cre^ mice that had received intraspinal injections of AAV.flex.eGFP was incubated in antibodies against pro‐CCK and NeuN (guinea pig antibody).

Sections were scanned with a Zeiss LSM 710 confocal microscope with Argon multiline, 405 nm diode, 561 nm solid state, and 633 nm HeNe lasers. Confocal image stacks were obtained through a 40× oil immersion lens (numerical aperture 1.3) with the confocal aperture set to 1 Airy unit. In each case, the entire mediolateral width of laminae I‐III was scanned to generate z‐series of at least 20 μm (and in many cases the full thickness of the section), with a z‐separation of 1 μm. Unless otherwise stated, for each antibody combination, two sections from each of three mice were selected for scanning. To avoid bias, this selection was made before the pro‐CCK‐immunoreactivity was viewed. Confocal scans were analyzed with Neurolucida for Confocal software (MBF Bioscience, Williston, VT). The lamina II‐III border was identified from the distribution of NeuN immunoreactivity, based on the relatively low neuronal packing density in lamina IIi. The lamina I‐II border was assumed to be 20 μm from the dorsal edge of the dorsal horn (Dickie et al., 2019). The lamina III/IV border was identified by the somewhat lower packing density of neurons in lamina IV (Molander, Xu, & Grant, 1984).

To determine the proportion of neurons in laminae I‐III that are pro‐CCK‐immunoreactive, we used a modification of the optical dissector method (Polgár, Gray, Riddell, & Todd, 2004) on sections from the three GRP::eGFP mice. The reference and look‐up sections were set 10 μm apart and initially only the NeuN and DAPI channels were viewed. All intervening optical sections were examined, and neuronal nuclei (NeuN+/DAPI+ structures) were selected if their bottom surface lay between the reference and look‐up sections. These were then plotted onto an outline drawing of the dorsal horn. The pro‐CCK channel was then switched on and the presence or absence of staining was determined for each selected neuron. These sections were also used to assess whether pro‐CCK was expressed by any GRP‐eGFP cells. For this analysis, we plotted the location of all pro‐CCK‐immunoreactive cells within the full depth of the confocal z‐series and then checked for the presence of eGFP in these cells.

To test whether any of the CCK cells were inhibitory interneurons, we identified pro‐CCK‐positive neurons in laminae I‐III through a depth of at least 24 μm in sections reacted with the Pax2 antibody from each of three mice. We then viewed the Pax2 channel and determined whether any of the selected cells had Pax2‐immunoreactive nuclei.

To look for evidence of coexpression of CCK and NKB, we analyzed sections reacted for pro‐CCK and the NKB precursor PPTB. Pro‐CCK‐immunoreactive neurons were identified throughout the full thickness of sections from three mice, and then the presence or absence of PPTB was noted.

To determine whether there was overlap between CCK and substance P populations, we analyzed sections from the three Tac1^Cre^ mice that had been injected with AAV.flex.eGFP. All pro‐CCK cells were identified throughout the full thickness of sections from three mice, and we then looked for the presence of eGFP (representing Tac1 expression) in these cells. Because we found some overlap between these populations, we then reversed the procedure by identifying all eGFP‐positive cells and determining whether they were pro‐CCK‐immunoreactive.

Sections reacted with antibodies against pro‐CCK, neurotensin, and PKCγ were used to look for co‐expression of pro‐CCK and neurotensin, and also to determine the relationship of both peptides to PKCγ. In this case, three sections from each of three mice were examined through their full thickness. Initially, all pro‐CCK cells were selected and these were then tested for the presence of neurotensin and PKCγ. In a separate analysis, we viewed the PKCγ channel and identified all of the neurons that showed strong or moderate levels of PKCγ immunoreactivity and had cell bodies that were completely contained within the section. We then switched on the pro‐CCK and neurotensin channels and recorded the presence or absence of these peptides in each of the selected PKCγ cells.

### 2.3 Fluorescent in situ hybridization histochemistry

Multiple‐labeling fluorescent in situ hybridization was performed with RNAscope probes and RNAscope fluorescent multiplex reagent kit 320850 (ACD BioTechne; Newark, CA). Fresh frozen lumbar spinal cord segments from three wild‐type mice was embedded in OCT mounting medium and cut into 12 μm thick transverse sections with a cryostat (Leica CM1850 or CM1860; Leica; Milton Keynes, UK). These were mounted non‐sequentially (such that sections on the same slide were at least four apart) onto SuperFrost Plus slides (48311‐703; VWR; Lutterworth, UK) and air‐dried. Reactions were carried out according to the manufacturer's recommended protocol. The probes used in this study, and the proteins/peptides that they correspond to, are listed in Table 2. Sections from three mice were incubated in the following probe combinations: (a) Cck, Slc17a6; (b) Prkcg, Cck, Trh; (c) Prkcg, Cck, Nts; (d) Tac1, Cck, Tac2; (e), Prkcg, Trh, Nts. In each case, the first listed probe was revealed with Alexa 488, the second with Atto 550, and the third with Alexa 647. Sections were mounted with Prolong‐Glass anti‐fade medium with NucBlue (Hoechst 33342) (ThermoFisher Scientific, Paisley, UK). Positive and negative control probes were also tested on other sections (Table 2).

**Table 2:** mRNAscope probes

Probe	Protein/peptide	Channel numbers	Catalogue numbers	*Z*‐pair number	Target region
Cck	CCK	1, 2	402271	12	23–679
Slc17a6	VGLUT2	2	319171	20	1,986–2,998
Prkcg	PKCγ	1	417911	20	685–2,438
Trh	TRH	2, 3	436811	20	128–1,280
Nts	Neurotensin	3	420441	20	2–1,188
Tac1	Substance P	1	410351	15	20–1,034
Tac2	NKB	3	446391	15	15–684
RNAscope multiplex positive control (Polr2a, Ppib, Ubc)	Polr2a: DNA‐directed RNA polymerase II subunit RPB1; Ppib: Peptidyl‐prolyl cis‐trans isomerase B; Ubc: Polyubiquitin‐C	1,2,3	320881	20 15 n/a	2,802–3,678 98–856 34–860
RNAscope multiplex negative control (dapB)	dapB: 4‐hydroxy‐tetrahydrodipicolinate reductase (derived from *Bacillus subtilis*)	1,2,3	320871	10	414–862

Sections were scanned with a Zeiss LSM 710 confocal microscope through the 40× oil‐immersion lens with the confocal aperture set to 1 Airy unit. In each case, a single optical plane through the middle of the section was scanned (Häring et al., 2018), using tile scanning to include the whole of laminae I‐IV. Five sections were scanned per animal except for the analysis of reactions (c) and (d) where three sections per animal were used. Sections were selected prior to viewing in situ hybridization florescence, to avoid bias.

Semiautomated analysis of transcript numbers per nucleus was conducted using the HALO FISH v1.1 module for RNAScope (Indica labs, Inc., Corrales). The locations of laminar boundaries were added to the images as described above, except that nuclear staining rather than NeuN was used to determine packing density. Recognition and segmentation of individual nuclei was performed based on NucBlue staining and an additional 1 μm perimeter was added to each nucleus to allow detection of perinuclear transcripts. This additional perimeter was omitted where cells were directly adjacent to each other. Any areas with poor nuclear segmentation were excluded manually from the analysis following examination of each segmented section.

Single RNA transcripts for each target gene appeared as individual puncta. Automatic transcript recognition was conducted with the HALO software at an analysis magnification of 1.2. Detection contrast and minimum intensity settings for each transcript were adjusted in real time until the segmented mark‐up accurately represented the confocal image. The number of transcripts per cell was derived from the product of the intensity and area of the transcript florescence within each nucleus and divided by a constant. This constant was initially set to the manufacturer's recommended value but was adjusted manually based on inspection of the derived data in several cells to ensure that the calculated transcript number accurately reflected that seen in the confocal image.

Data output following analysis consisted of manual inspection of the section to ensure accuracy, followed by export of a table containing each individual cell's transcript numbers. This was further analyzed in Microsoft Excel. Cells were defined as positive for expression of a given gene if they contained greater than four transcripts. Colocalization of expression in cells was quantified by generating pivot contingency tables.

### 2.4 Antibody characterization

Details of the primary antibodies that were used in this study are given in Table 1. Specificity of the pro‐CCK antibody has been demonstrated by showing that it stains identical cells to those detected by fluorescent in situ hybridization with a probe against CCK mRNA in mouse cortex and hippocampus (Booker et al., 2017). The mouse monoclonal antibody NeuN, which was generated against cell nuclei extracted from mouse brain, was found to react with a protein specific to neurons (Mullen, Buck, & Smith, 1992), and this has subsequently been identified as the splicing factor Fox‐3 (Kim, Adelstein, & Kawamoto, 2009). We have found that the guinea‐pig NeuN antibody stains the same cells as the mouse antibody (Dickie et al., 2019). Staining with the neurotensin antibody is identical to that seen with a well‐characterized rabbit neurotensin antibody, and is blocked by preincubation with 10^−4^ M neurotensin (Porteous et al., 2011). The PKCγ antibody recognizes a band of the appropriate molecular weight on western blots from wild‐type, but not PKCγ^−/−^ mice (Yoshida et al., 2006). The PPTB antibody recognizes PPTB, but not PPTA, NKB, or substance P on dot blots, and staining is blocked by preincubation with the immunizing peptide. The Pax2 antibody recognizes bands of the appropriate molecular weight on western blots of mouse embryonic kidney (Dressler & Douglass, 1992). Staining with the eGFP antibody shows an identical distribution to that of the native fluorescent protein in tissues.

### 2.5 Specificity of fluorescent in situ hybridization

All neurons were labeled with the positive control probes (which detect mRNAs for ubiquitously expressed proteins), while none were labeled with the negative control probe (which detects a bacterial enzyme).

### 2.6 Figures

Figures were composed with Adobe Photoshop (CS6). Image brightness and contrast were adjusted using the levels setting.

## 3 RESULTS

### 3.1 Expression of CCK by excitatory neurons in the dorsal horn

**Figure 1:**
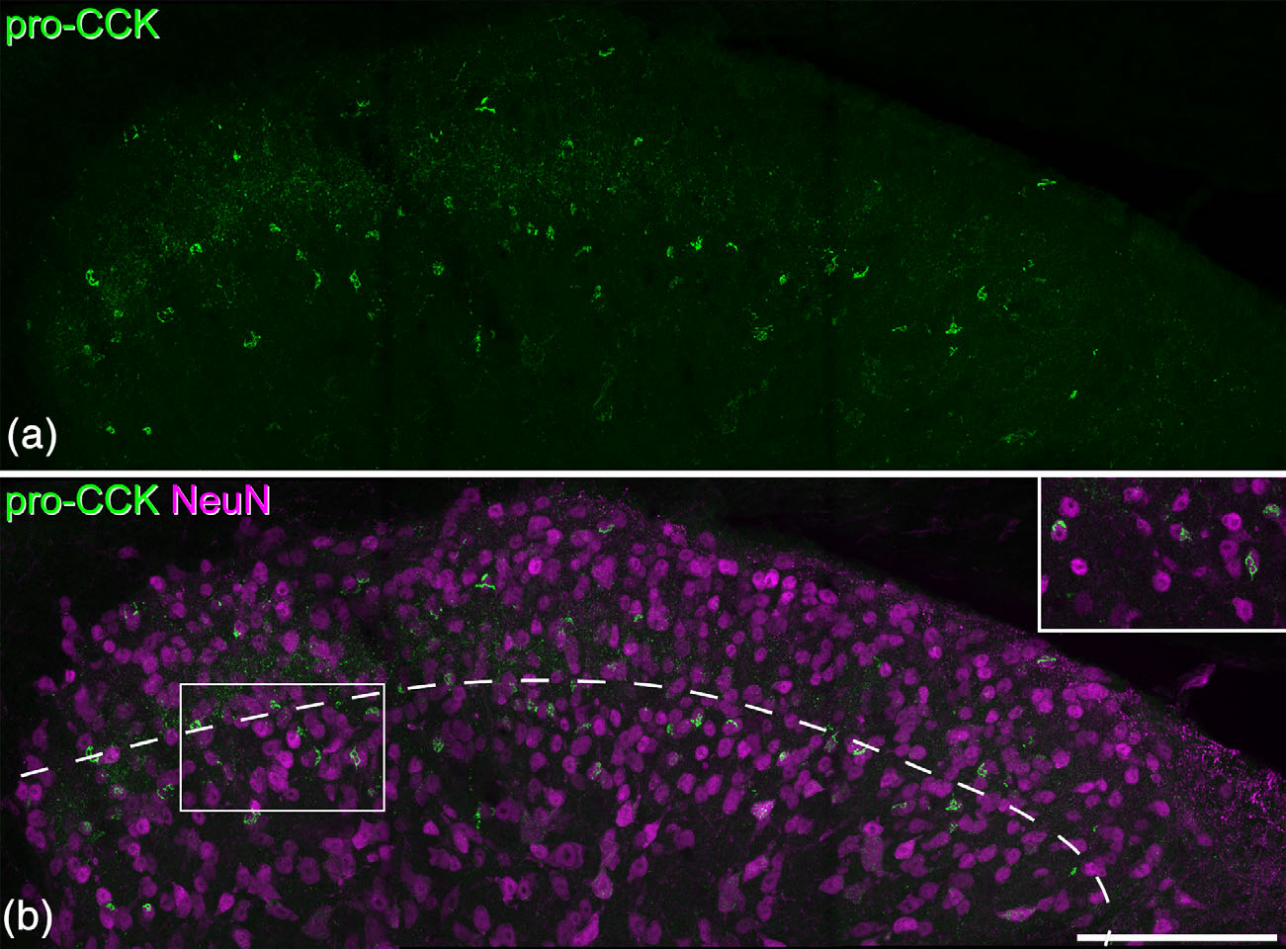
Pro‐cholecystokinin (CCK) immunoreactivity in the mouse dorsal horn. (a) A maximum intensity projection of 27 confocal optical sections (1 μm z‐separation) showing immunostaining with the pro‐CCK antibody in the dorsal horn. Brightly labeled profiles represent perikaryal staining. (b) The same field showing both NeuN (magenta) and pro‐CCK (green). The dashed line shows the approximate position of the lamina II/III border. The rectangle indicates the region shown in the inset. Pro‐CCK‐immunoreactive cells are scattered throughout laminae I‐II, but are particularly numerous in lamina III. Inset: The relationship between pro‐CCK and NeuN staining can be clearly seen in a maximum intensity projection of three optical sections. Scale bar = 100 μm [Color figure can be viewed at wileyonlinelibrary.com]

**Figure 2:**
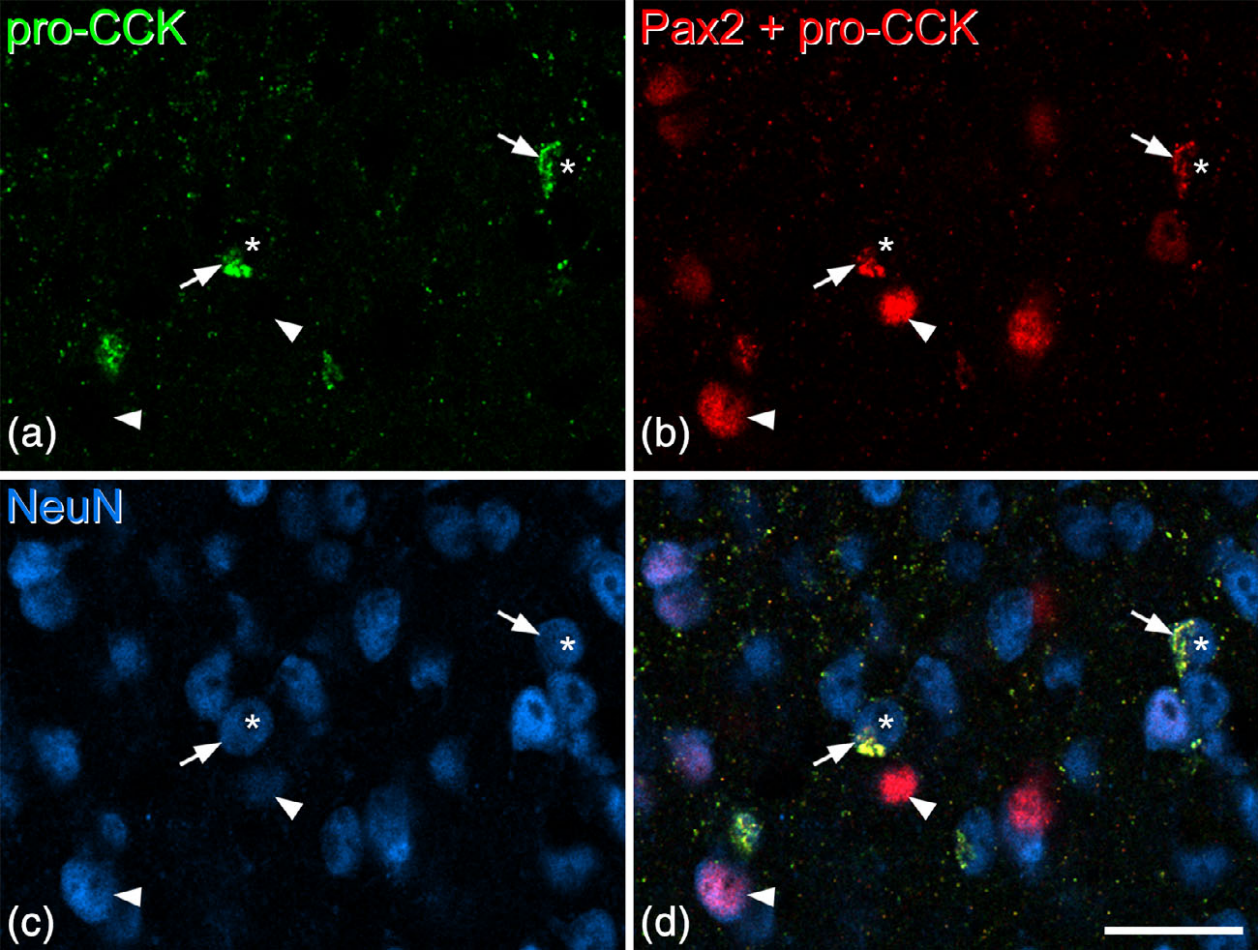
Lack of Pax2 immunoreactivity in the nuclei of pro‐cholecystokinin (CCK)‐positive cells. These images are from a section that had been reacted with rabbit pro‐CCK antibody (revealed with Alexa 488) and mouse NeuN antibody (revealed with Alex 647). The section was subsequently reacted with Pax2 antibody (also raised in rabbit), which was revealed with Rhodamine Red. (a) Staining in the Alexa 488 channel is restricted to pro‐CCK‐positive structures, which represent perikaryal cytoplasm of CCK‐expressing neurons. (b) Staining in the Rhodamine channel detects both pro‐CCK and Pax2, because the Rhodamine‐conjugated secondary anti‐rabbit antibody was applied after both rabbit primary antibodies (pro‐CCK and Pax2). However, these can readily be distinguished because the Pax2 profiles are not labeled with Alexa 488 and because Pax2 staining is nuclear, whereas pro‐CCK is cytoplasmic. (c) The same field scanned to reveal NeuN. (d) The merged image shows pro‐CCK‐immunoreactivity in the perikaryal cytoplasm of cells (two marked with arrows) that have nuclei that are negative for Pax2 (asterisks). Two Pax2‐positive cells, which lack pro‐CCK, are indicated with arrowheads. All images are from a single optical section. Scale bar = 20 μm [Color figure can be viewed at wileyonlinelibrary.com]

In transverse sections immunostained for pro‐CCK, labeling was seen in the perikaryal cytoplasm of neurons. These immunoreactive neurons were particularly numerous in the deep dorsal horn (laminae III‐V), and were scattered at lower density in laminae I and II (Figure 1). Quantitative results obtained with the disector method from three mice are shown in Table 3. Pro‐CCK‐immunoreactive cells accounted for ~5% of all neurons in laminae I‐II and 15% of those in lamina III. To test whether CCK cells in laminae I‐III were excitatory or inhibitory, we compared Pax2 and pro‐CCK immunostaining in sections from three wild‐type mice. Altogether, 247 pro‐CCK cells were identified (76–95 per mouse) and none of these had Pax2 immunoreactivity in the nucleus (Figure 2), indicating that they are all excitatory neurons. We have previously determined the proportions of neurons in laminae I‐III of the mouse dorsal horn that lack immunoreactivity for GABA and glycine, and are therefore presumably excitatory neurons (Polgár et al., 2013). Based on these results, we estimate that pro‐CCK can be detected in 6.9, 6.7, and 24.7% of the excitatory neurons in laminae I, II, and III, respectively (Table 3). Laminae I‐II are often considered together as the superficial dorsal horn, and pro‐CCK‐immunoreactive cells would account for 6.8% of the excitatory neurons in this region.

**Table 3:** Percentages of neurons in laminae I‐III that were pro‐CCK‐immunoreactive

	Number of neurons counted	Pro‐CCK‐immunoreactive cells	% of neurons that were pro‐CCK‐immunoreactive	% of neurons that are GABA/glycine‐negative^a^	Estimated % of excitatory neurons that contain pro‐CCK
I	101 (72–116)	5 (3–8)	4.8 (3.5–6.9)	69.5	6.9
II	284.3 (207–339)	15 (9–26)	5.1 (3.3–7.7)	75.8	6.7
III	236.7 (187–278)	36.3 (28–46)	15.4 (12.6–18.8)	62.4	24.7

In each case, the mean values for the three mice are shown, with the range in brackets.

a The percentages of neurons in each lamina that lack immunoreactivity for GABA and glycine are taken from four wild‐type C57BL/6 mice examined by Polgár et al. (2013).

**Figure 3:**
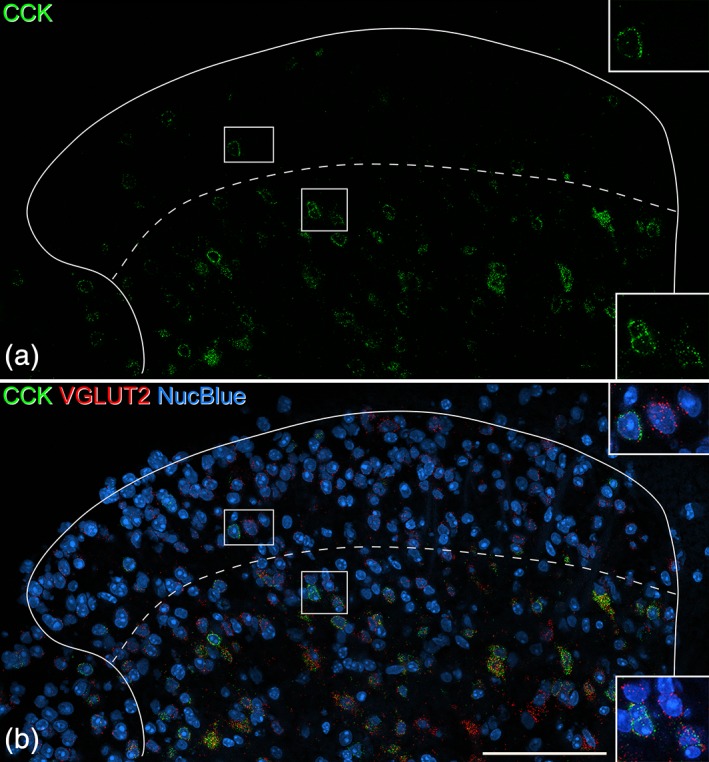
In situ hybridization for cholecystokinin (CCK) and VGLUT2 in the dorsal horn. The section has been reacted with probes for the Cck and Slc17a6 (VGLUT2) mRNAs and counterstained with NucBlue to reveal nuclei. The dashed line indicates the approximate position of the lamina II‐III border. (a) There are relatively few CCK‐positive cells in the superficial dorsal horn (laminae I‐II), but these are very numerous in deeper laminae. (b) When labeling for both mRNAs is viewed together with staining for nuclei, it can be seen that there are numerous VGLUT2‐positive cells throughout the dorsal horn, and that many of these (particularly below the lamina II‐III border) are CCK‐positive. Insets show the boxed regions at higher magnification. Each of these contains CCK‐positive cells that are also labeled with the VGLUT2 probe. All images are from a single optical section. Scale bar = 100 μm [Color figure can be viewed at wileyonlinelibrary.com]

Quantitative results for the in situ hybridization analysis of VGLUT2 and CCK in sections processed with probes directed against Slc17a6 (VGLUT2) and Cck mRNAs are shown in Table 4, and an example of the labeling is shown in Figure 3. As expected, the vast majority of cells that were positive for Cck mRNA also contained VGLUT2 mRNA (93 and 98% for laminae I‐II and lamina III, respectively). These accounted for 7% of the VGLUT2+ cells in laminae I‐II and for 36% of those in lamina III. Although we did not formally analyze the dorsal horn below lamina III, we also noted that ~30% of the VGLUT2+ cells in the region corresponding to lamina IV were also CCK+.

**Table 4:** In situ hybridization analysis for CCK and VGLUT2

	Number of neurons with VGLUT2 mRNA	Number of neurons with CCK mRNA	% of CCK neurons with VGLUT2	% of VGLUT2 neurons with CCK
I‐II	772 (714–848)	60.0 (57–64)	93.3% (91.2–94.9%)	7.3% (6.1–8%)
III	404.7 (332–461)	148.3 (125–173)	97.6% (96.5–98.6%)	35.9% (34.9–36.7%)

In each case, the mean values for the three mice are shown, with the range in brackets.

The in situ hybridization analysis was based on profile counts (rather than stereology) and there may therefore be a bias toward larger neurons, which are more likely to have been included in the optical section analyzed (Guillery, 2002). However, the close agreement between the estimates of excitatory neurons that are CCK‐positive in the superficial dorsal horn (6.8% with immunocytochemistry, 7.3% with in situ hybridization) suggests that this did not have an impact on our estimates in this region. The proportion of lamina III excitatory neurons in which Cck mRNA was detected was somewhat higher than our estimate of the proportion that were pro‐CCK‐immunoreactive (36% compared to 25%). This discrepancy could have resulted from a size bias, but may have been due to our failure to detect pro‐CCK in some of the CCK‐expressing neurons in this lamina.

### 3.2 CCK is not expressed by PPTB‐positive or GRP‐eGFP neurons

**Figure 4:**
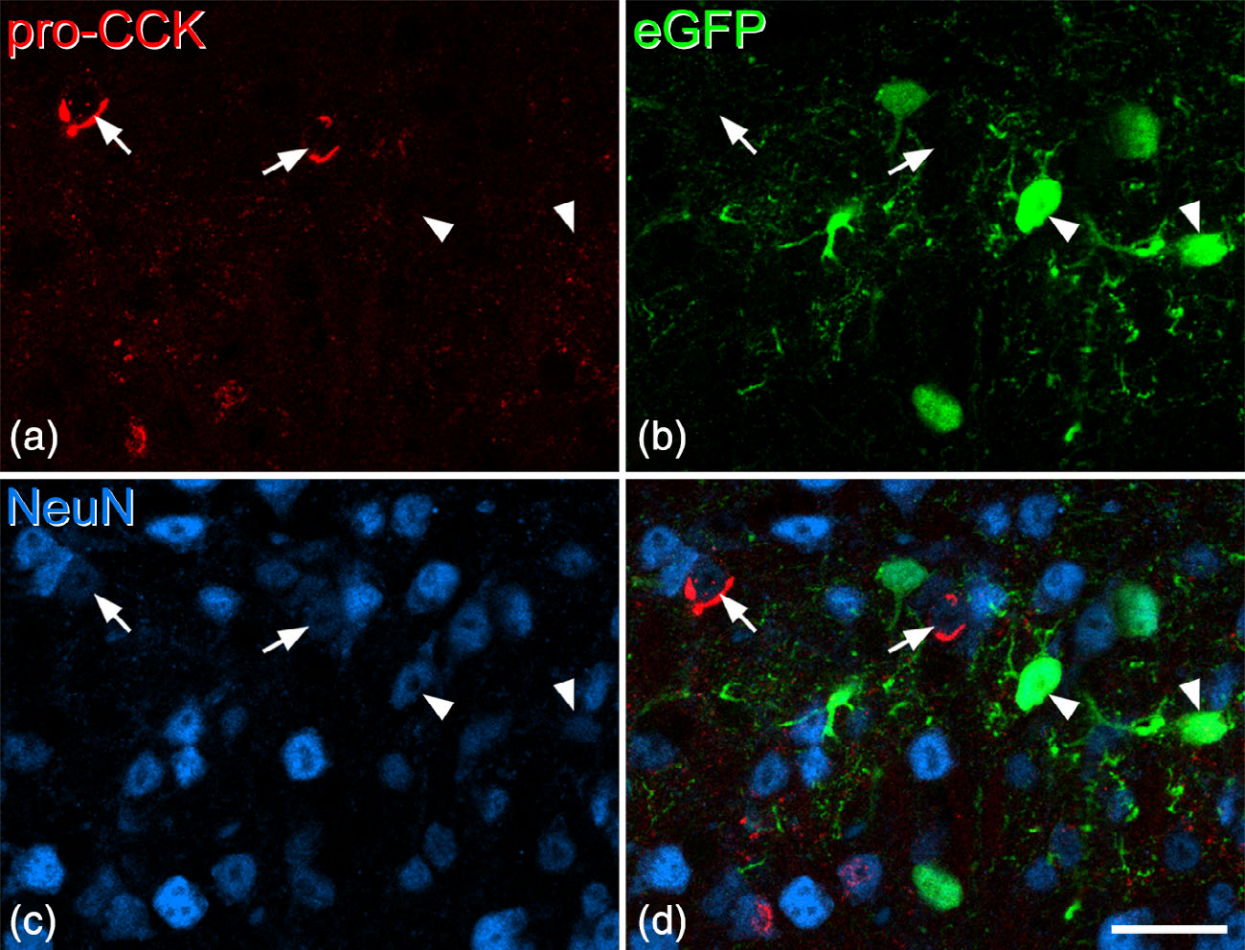
Lack of colocalization of pro‐cholecystokinin (CCK) and enhanced green fluorescent protein (eGFP) in tissue from the GRP::eGFP mouse. (a–c) Scans from the superficial dorsal horn to show immunostaining for pro‐CCK, eGFP, and NeuN. (d) The merged image reveals that pro‐CCK and eGFP are present in different neuronal populations. Two of the pro‐CCK cells are indicated with arrows, while two eGFP‐positive cells are marked with arrowheads. Images are from three optical sections at 1 μm z‐separation. Scale bar = 20 μm [Color figure can be viewed at wileyonlinelibrary.com]

In the sections from the three GRP::eGFP mice, we identified an average of 129 (116–152) pro‐CCK‐immunoreactive cells in laminae I‐III and all except two of these cells (both located in lamina III) were eGFP‐negative (Figure 4), indicating that CCK is very seldom expressed in GRP‐eGFP neurons.

**Figure 5:**
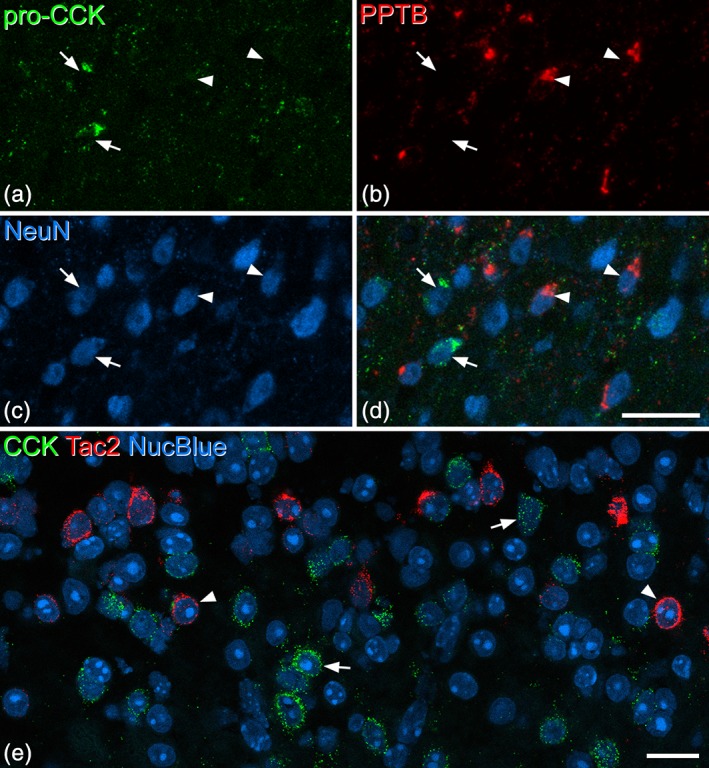
Lack of colocalization of pro‐cholecystokinin (CCK) and the neurokinin B precursor preprotachykinin B (PPTB) in the dorsal horn. (a–c) Scans from lamina II and III of a section that had been reacted with antibodies against pro‐CCK, PPTB, and NeuN. (d) The merged image reveals that pro‐CCK and PPTB are present in different neuronal populations. Two of the pro‐CCK cells are indicated with arrows, while two PPTB‐positive cells are marked with arrowheads. Images are from four optical sections at 1 μm z‐separation. (e) A single confocal optical plane from a section that had been reacted with probes against CCK and Tac2 mRNAs, and counterstained with NucBlue. This field shows the region on either side of the lamina II‐III border. Many cells are positive for either CCK mRNA (green, two marked with arrows) or Tac2 mRNA (red, two marked with arrowheads). Scale bars for (a–d) and for (e) = 20 μm [Color figure can be viewed at wileyonlinelibrary.com]

In sections from three wild‐type mice that had been immunoreacted for PPTB and pro‐CCK, we identified a mean of 95 pro‐CCK‐immunoreactive cells (range 80–115) in laminae I‐III in sections from three mice, and found that none of these cells were PPTB‐immunoreactive (Figure 5a–d). In situ hybridization showed minimal overlap: in sections from three mice that were reacted with probes against Cck and Tac2 mRNA, we identified an average of 101 (range 90–113) Tac2 mRNA cells and 170 (109–235) Cck mRNA cells in laminae I‐III per animal. Only 1.6% (1–2%) of the Tac2 cells were Cck+, while 1% (0.9–1.2%) of the Cck + cells were Tac2+ (Figure 5e).

### 3.3 Partial overlap between CCK and substance P populations

**Figure 6:**
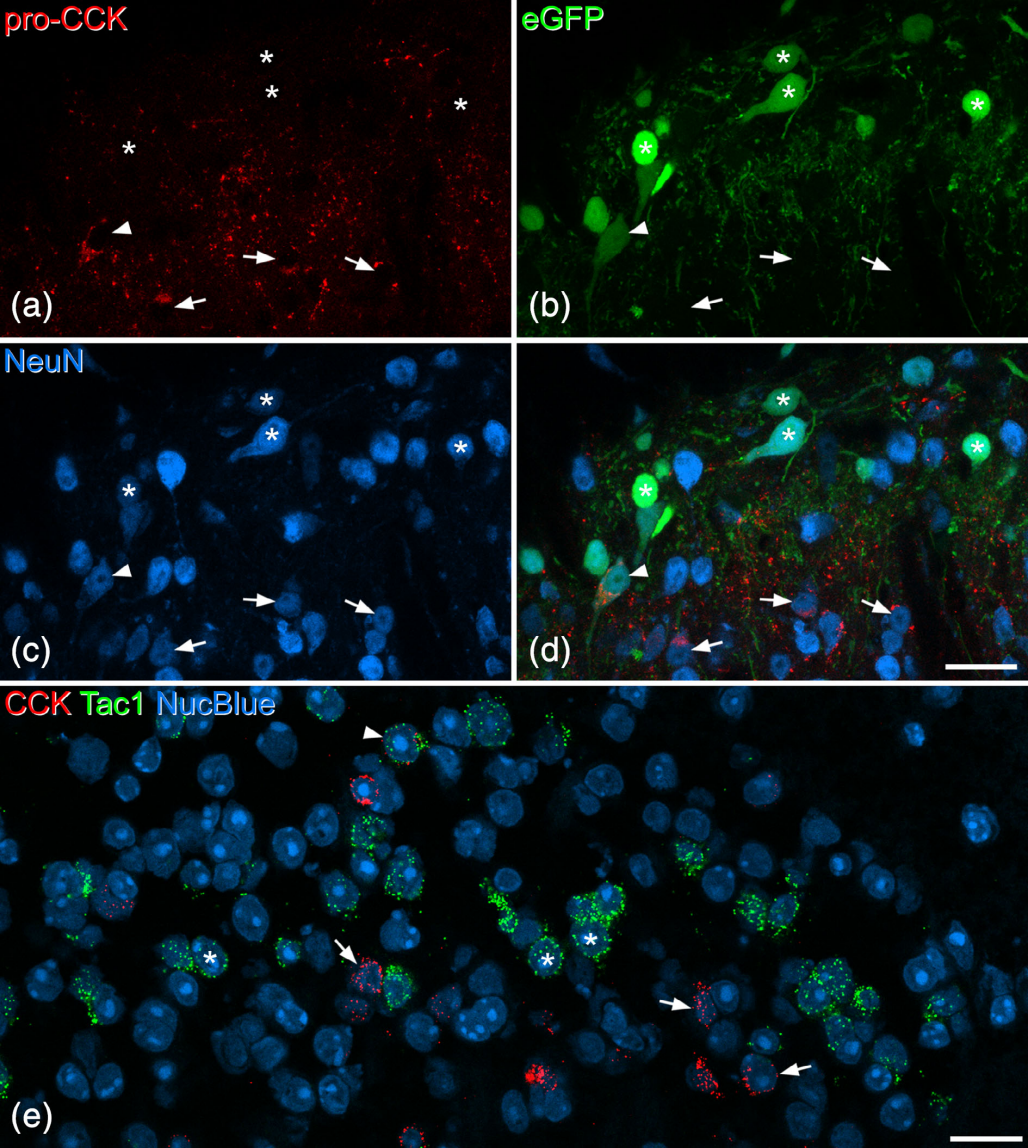
Limited coexpression of cholecystokinin (CCK) and substance P in the dorsal horn. (a–d) Part of the superficial dorsal horn from a Tac1^Cre^ mouse that had been injected with AAV.flex.eGFP 8 days prior to perfusion fixation. (a–c) Show immunostaining for pro‐CCK, enhanced green fluorescent protein (eGFP) fluorescence, and NeuN immunoreactivity, while (d) shows a merged image. Numerous eGFP‐positive (substance P‐expressing) cells can be seen in the upper part of the field (which corresponds to laminae I‐II), and most of these lack pro‐CCK (some are indicated with asterisks). There are several pro‐CCK‐positive (eGFP‐negative) cells in the lower part of the field, some marked with arrows. A double‐labeled cell is marked with an arrowhead. Images are from four optical sections at 1 μm z‐spacing. (e) A single confocal optical plane from a section that had been reacted with probes against CCK and Tac1 mRNAs, and counterstained with NucBlue. The field of view contains numerous Tac1 mRNA‐positive cells (three marked with asterisks), which form a dense band across the middle of lamina II. There are also several CCK mRNA‐positive cells (three shown with arrows), and most of these are located more ventrally. A single cell with both Tac1 and CCK mRNAs is indicated with an arrowhead. Scale bars for (a–d) and for (e) = 20 μm [Color figure can be viewed at wileyonlinelibrary.com]

**Figure 7:**
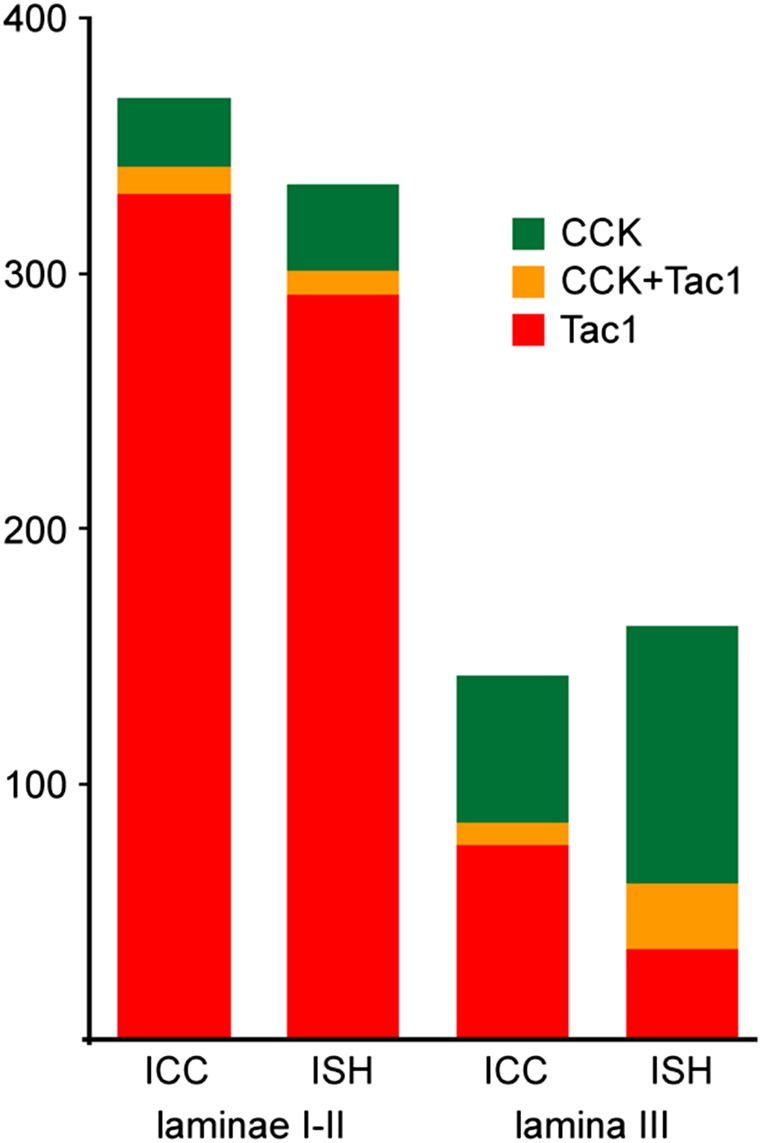
Quantitative data for coexpression of cholecystokinin (CCK) and substance P in the dorsal horn. The bar chart shows the mean numbers of cells in laminae I‐II and lamina III that were defined as expressing CCK, Tac1 (the gene coding for substance P), or both peptides, based on immunocytochemistry (ICC) or in situ hybridization (ISH). The immunocytochemical detection of pro‐CCK was performed on 3 Tac1^Cre^ mice that had received intraspinal injections of AAV.flex.eGFP, and eGFP‐containing cells were defined as Tac1‐positive. In situ hybridization was performed with RNAscope and single optical sections from three wild‐type mice were analyzed. Cells containing four or more transcripts for CCK or Tac1 mRNAs were defined as positive for the respective peptide. For further details, see Table 5 [Color figure can be viewed at wileyonlinelibrary.com]

In sections from Tac1^Cre^ mice that had been injected with AAV.flex.eGFP, we found a similar distribution of eGFP‐labeled (Tac1‐expressing) cells to that described previously (Dickie et al., 2019; Gutierrez‐Mecinas et al., 2017). These were concentrated in laminae I and II, with labeled cells scattered through the deeper dorsal horn. Although most pro‐CCK‐immunoreactive cells were different from those that were eGFP‐labeled, we found some overlap. In laminae I‐II, double‐labeled neurons corresponded to 29% of the pro‐CCK‐immunoreactive cells, and 3% of those that were eGFP‐labeled. In lamina III, the corresponding proportions were 13 and 10%, respectively (Table 5, Figures 6a–d and 7). When we compared the distribution of Cck and Tac1 mRNAs in wild‐type mice, the results for laminae I‐II were similar: double‐labeled cells corresponded to 21% of those with Cck and 3% of those with Tac1 mRNA (Table 5, Figures 6e and 7). However, in lamina III we found a considerably higher degree of overlap, with double‐labeled cells corresponding to 21% of those with Cck and 40% of those with Tac1 mRNA (Table 5, Figures 6e and 7). The much higher proportion of Tac1+ cells with Cck mRNA in lamina III may have resulted from our failure to detect pro‐CCK‐immunoreactivity in some CCK‐expressing cells in this region (see above).

**Table 5:** Extent of overlap of CCK and Tac1 populations

		Number of Tac1+ neurons	Number of CCK+ neurons	Number of double‐labeled neurons	Double‐labeled neurons as % of Tac1 neurons	Double‐labeled neurons as % of CCK neurons
Lamina I‐II	Immunocytochemistry	342.3 (274–398)	37.7 (35–39)	11 (10–12)	3.3 (2.8–4.4)	29.2 (28.2–30.8)
	In situ hybridization	301.3 (280–324)	43.7 (43–45)	9.3 (9–10)	3.1 (2.8–3.3)	21.4 (20.9–22.2)
Lamina III	Immunocytochemistry	85 (68–105)	66.7 (59–71)	8.7 (3–13)	9.7 (4.4–12.4)	13.2 (4.3–18.3)
	In situ hybridization	61.0 (50–74)	126.3 (64–192)	25.3 (16–39)	40.1 (32.0–52.7)	20.8 (17.1–25.0)

In each case, the mean values for the three mice are shown, with the range in brackets. For the immunocytochemical analysis of Tac1‐expressing neurons, we counted eGFP+ cells in sections from Tac1^Cre^ mice that had received intraspinal injections of AAV.flex.eGFP.

### 3.4 Relationship of CCK to neurotensin, PKCγ, and TRH

**Figure 8:**
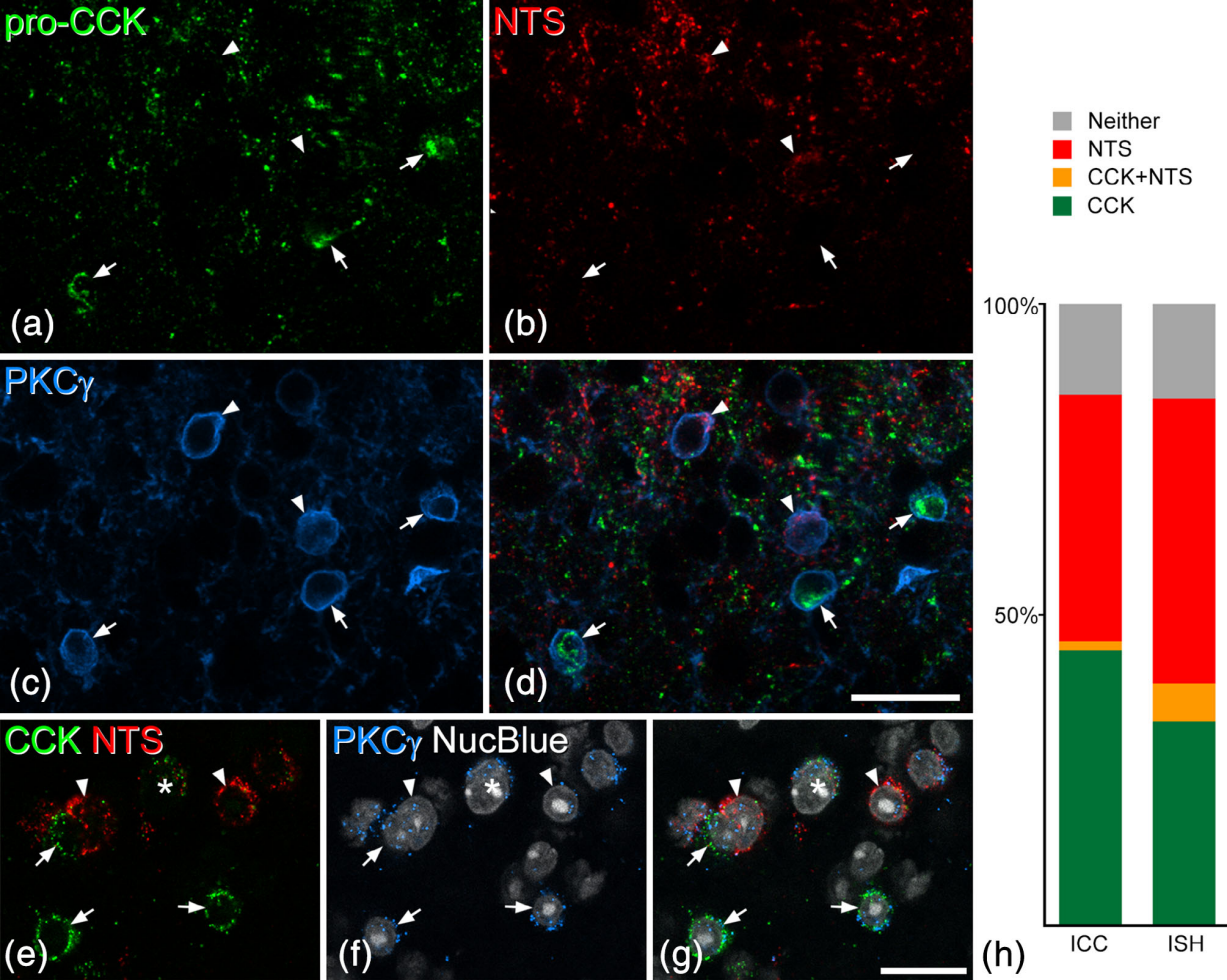
The relationship of pro‐cholecystokinin (CCK) and neurotensin to protein kinase Cγ (PKCγ) in the dorsal horn. (a–c) A projection of three optical sections (1 μm z‐separation) through the lamina II‐III border region scanned to reveal immunoreactivity for pro‐CCK, neurotensin (NTS), and PKCγ. Cell bodies of five neurons with strong to moderate PKCγ staining are visible. (d) The merged image shows that two of these are neurotensin‐immunoreactive (arrowheads), while the other three are pro‐CCK‐immunoreactive (arrows). In each case, immunostaining for the neuropeptide/precursor can be seen in the perikaryal cytoplasm just inside the ring of PKCγ‐immunostaining, which represents the plasma membrane. (e–g) In situ hybridization in a section that had been reacted with probes for CCK, neurotensin, and PKCγ mRNAs and counterstained with NucBlue. Several PKCγ+ cells are present in this field, and six of these are marked. Three (arrows) are positive for CCK mRNA and two (arrowheads) for neurotensin mRNA, while one (asterisk) contains mRNAs for both CCK and neurotensin. The in situ hybridization images were obtained from a single optical section. (h) Quantitative analysis of the proportions of PKCγ cells that were positive for CCK and/or NTS detected with either immunocytochemistry (ICC) or in situ hybridization (ISH). The bar chart shows the results for cells with strong to moderate PKCγ‐immunoreactivity (ICC) or that had 10 or more Prkcg mRNA transcripts (ISH). In each case, these have been divided into those that were positive for CCK only (green), neurotensin only (red), or both CCK and neurotensin (orange). Cells that were negative for both peptides are represented as gray bars. In each case, data from three animals were pooled. Scale bars for (a–d) and for (e‐g) = 20 μm [Color figure can be viewed at wileyonlinelibrary.com]

In sections from three wild‐type mice that were immunoreacted for pro‐CCK, neurotensin, PKCγ, and NeuN, we identified a mean of 151.3 (range 125–185) pro‐CCK‐immunoreactive neurons in laminae I‐III and found that only 0.9% (0.7–1.1%) of these were neurotensin‐immunoreactive. However, 47.1% (41.1–51.4%) of the pro‐CCK cells were PKCγ‐positive and these cells generally showed strong immunostaining for PKCγ (Figure 8a–d).

Because we had previously found that many neurotensin‐expressing cells also showed moderate‐strong labeling for PKCγ (Gutierrez‐Mecinas et al., 2016), we examined the relationship between neurotensin and CCK among PKCγ‐expressing cells. For this analysis, we excluded cells that showed weak PKCγ‐immunoreactivity, because we have reported that many of these are PPTB‐immunoreactive (Gutierrez‐Mecinas et al., 2016), and PPTB shows minimal coexpression with either CCK or neurotensin. We identified a total of 838 cells with moderate‐strong PKCγ‐immunoreactivity in sections from three mice, and found that 44% of these were pro‐CCK+ only, 40% were neurotensin+ only, while 2% had both types of peptide immunoreactivity, and 15% had neither (Figure 8a–d,h). This suggests that the great majority of dorsal horn neurons that have significant levels of PKCγ express either CCK or neurotensin. To provide further evidence for this, we also carried out in situ hybridization for Cck, Nts, and Prkcg mRNAs and performed a similar analysis. In this case, we set the threshold at 10 Prkcg mRNA transcripts per cell in an attempt to exclude neurons that expressed low levels of PKCγ. We identified 131 cells that met this criterion in sections from three mice, and found that 33% of these cells were positive for Cck mRNA only, 46% were positive for Nts mRNA only, while 6% were positive for both peptide mRNAs, and 15% were negative for both (Figure 8e–h).

**Figure 9:**
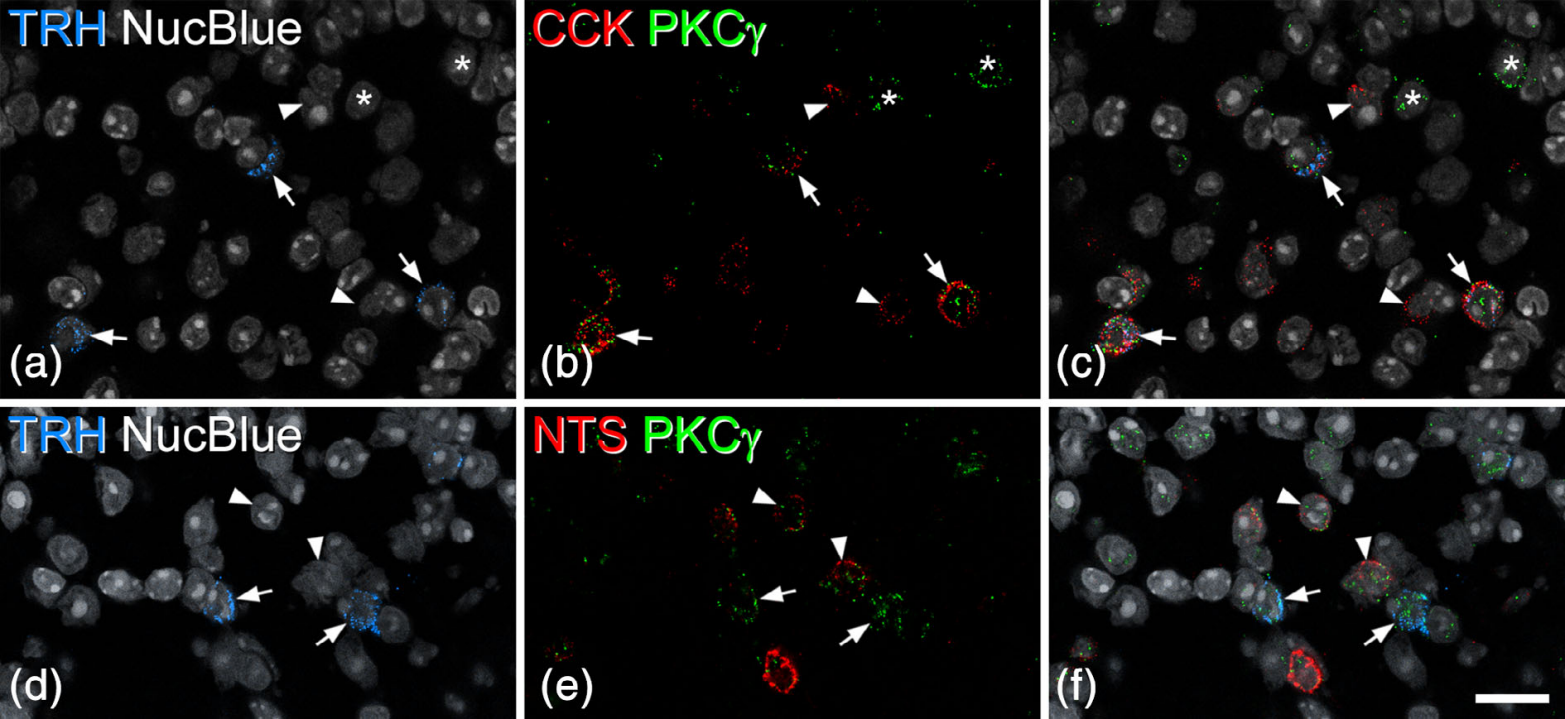
The relationship of thyrotropin‐releasing hormone (TRH)‐expressing dorsal horn neurons to cholecystokinin (CCK), protein kinase Cγ (PKCγ), and neurotensin. (a–c) A scan through laminae II and III from a section reacted with probes against TRH, CCK, and PKCγ mRNAs, and counterstained with NucBlue. Three TRH+ cells are marked with arrows, and all of these are positive for both CCK and PKCγ mRNAs. Asterisks indicate two PKCγ cells that lack message for TRH and CCK, while the arrowheads show two CCK cells that are negative for TRH and PKCγ. (d–f) A scan through the equivalent region from a section reacted with probes against TRH, neurotensin, and PKCγ mRNAs, and counterstained with NucBlue. Two TRH+ cells that contain mRNA for PKCγ, but not for neurotensin, are indicated with arrows. The arrowheads point to two cells that are positive for neurotensin and PKCγ mRNA, but negative for TRH mRNA. All images were obtained from single optical sections. Scale bar = 20 μm [Color figure can be viewed at wileyonlinelibrary.com]

TRH‐expressing cells were detected with in situ hybridization, and their distribution was similar to that reported previously (Allen Spinal Cord Atlas, http://mousespinal.brain-map.org/). The cells were relatively sparse, and were present in the inner part of lamina II and the dorsal part of lamina III (Figure 9a,d). On average, 29.7 (22–35) Trh mRNA‐positive cells were identified in sections from three mice that had been reacted with probes against Trh, Cck, and Prkcg mRNAs, and 85% (81–89%) of these cells were Cck mRNA‐positive (Figure 9a–c). Because the Trh mRNA+ cells represent a very small proportion of CCK‐expressing cells we did not attempt to determine the proportion of all CCK cells that expressed TRH. However, in a band extending 40 μm either side of the lamina II‐III border we identified between 86 and 103 CCK mRNA+ cells, and found that 25% (21–28%) of these were TRH‐positive. In these sections, Trh mRNA was present in 46% (42–53%) of those CCK+ cells that had 10 or more Prkcg mRNA transcripts. To further assess the relationship between TRH and PKCγ, we analyzed sections reacted with this probe combination, and also sections reacted with probes against Prkcg, Trh, and Nts mRNAs. We identified 60.7 (58–64) Trh mRNA‐positive cells, and found that 91% (89–92%) of these were positive for Prkcg mRNA (i.e., had four or more transcripts; Figure 9). The relationship between TRH and neurotensin was examined in sections reacted with the latter probe combination. We identified 31 (26–38) Trh mRNA+ cells and 138.7 (131–152) Nts mRNA+ cells in these sections. We found limited co‐expression, as only 4% (2.2–5.3%) of the Nts mRNA+ cells had Trh mRNA, while 18% (12–21%) of Trh mRNA+ cells contained Nts mRNA (Figure 9d–f).

## 4 DISCUSSION

Our main findings were: (a) that CCK is expressed in <10% of excitatory neurons in the superficial dorsal horn, but in a far higher proportion of those in deeper laminae; (b) that these cells are largely separate from those that express NKB or neurotensin, as well as from the population defined by expression of eGFP in the GRP::eGFP mouse; (c) that there is limited overlap with cells that express substance P; (d) that many of the CCK cells express PKCγ, and together with neurotensin cells, these account for ~85% of the cells with moderate or high levels of PKCγ expression; and (e) that TRH is restricted to a small subset of the CCK cells, accounting for around half of those that express PKCγ.

### 4.1 Technical considerations

We used two different approaches as surrogate measures for gene expression in dorsal horn neurons: in situ hybridization with RNAscope that detects mRNA transcript numbers, and immunocytochemistry, which reveals the proteins or peptides that are generated from these mRNAs. Although the levels of mRNAs and their corresponding proteins are correlated within individual cells, there is not a direct relationship between them, for example due to variations in post‐transcriptional regulation and protein stability (Vogel & Marcotte, 2012). It is, therefore, not possible to predict how much mRNA in a particular neuron will lead to a specific level of protein expression in that cell. Comparing these two techniques, the levels of protein or peptide, as detected by immunocytochemistry, are presumably more important in terms of function. However, knowledge of mRNA production forms the basis for transcriptomic methods to define neuronal populations (Häring et al., 2018), and also provides valuable information that is needed for molecular/genetic approaches in which specific neurochemical populations are to be targeted.

Although it is possible to combine immunocytochemistry with in situ hybridization, and therefore confirm expression in the same cell (Booker et al., 2017), we did not attempt to do this in the present study, as we wished to retain optimal sensitivity offered by the RNAscope technique. Nonetheless, the consistency between patterns of colocalization that we observed with the two techniques strongly suggests that in general, they were detecting equivalent populations. However, as the RNAscope method allows identification of individual mRNA molecules, it is likely to reveal cells with very low levels of gene expression, which may be missed with immunocytochemistry.

### 4.2 Neurochemical populations among dorsal horn excitatory interneurons

Classification schemes for dorsal horn neurons have been based mainly on the laminar location, morphology, electrophysiological properties, and neurochemistry of the cells (Abraira et al., 2017; Graham, Brichta, & Callister, 2007; Grudt & Perl, 2002; Yasaka, Tiong, Hughes, Riddell, & Todd, 2010). Among the four neurochemical populations that we had previously identified, the neurotensin and NKB cells had certain features in common, as they were both concentrated on either side of the lamina II‐III border, and both included cells that expressed PKCγ. We have recently provided evidence that the other two populations differed from each other in both morphological and electrophysiological properties with those that express substance P corresponding to radial cells (which generally show delayed firing) and the GRP‐GFP cells resembling a population that had been defined by Grudt and Perl (2002) as “transient central cells” (Dickie et al., 2019).

Häring et al. (2018) used clustering of transcriptomic data to define 15 populations of excitatory neuron, which they named Glut1‐15. Their analysis generally supports our earlier findings, by showing that mRNAs for substance P, neurotensin, and NKB are highly expressed by different populations: Glut10‐11 for substance P, Glut4 for neurotensin, and Glut5‐6 for NKB. However, both of their substance P populations (Glut10 and 11) include many cells in deeper laminae, and therefore extend beyond the lamina II radial cell population that we have identified (Dickie et al., 2019). Häring et al. did not define a population specifically associated with GRP expression, and they reported that GRP mRNA was present in cells belonging to several of their populations (Glut5‐12). Although virtually all eGFP‐expressing cells in the GRP‐eGFP mouse contain GRP mRNA, many cells with GRP mRNA do not express GFP in this line (Dickie et al., 2019; Solorzano et al., 2015), and this suggests that eGFP is restricted to a subset of the GRP‐expressing cells. We have found that at least some of these GRP‐eGFP cells contain mRNA for the neuromedin U receptor 2 (Nmur2; AMB, unpublished data), and these may therefore correspond to the Glut8 population, in which Nmur2 mRNA is enriched.

Häring et al. (2018) assigned CCK‐expressing neurons to three separate clusters: Glut1, 2, and 3. These differed somewhat in laminar location, with cells belonging to Glut2 being scattered through laminae I‐II, those in Glut3 forming a compact band in laminae IIi‐III, and those in Glut1 (the largest population) being virtually restricted to the deep dorsal horn (laminae III‐V). In addition, expression of TRH was limited to the Glut3 population. In the present study, we found a relatively small number of CCK+ cells in laminae I‐II, and most of these are likely to belong to the Glut2 population of Häring et al. The more numerous CCK‐expressing cells that were distributed throughout the deeper laminae presumably correspond mainly to the Glut1 and Glut3 populations, with the subset that coexpressed TRH representing the Glut3 cells. The very limited overlap between pro‐CCK‐immunoreactive cells and those that were neurotensin‐ or PPTB‐immunoreactive is consistent with the results of Häring et al., and provides further evidence that these neuropeptides are expressed in different populations of excitatory interneurons. We did see a moderate degree of overlap between Tac1 and CCK, with around a quarter of CCK+ cells in laminae I‐II being Tac1‐positive. However, these only accounted for 3% of Tac1 cells, indicating that CCK is seldom, if ever, expressed by substance P radial cells in lamina II (Dickie et al., 2019). The cells that express both CCK and Tac1 in this region could belong to either Glut9 or Glut10, because both peptides are significantly expressed in these clusters, and they include cells in the superficial dorsal horn. CCK and Tac1 showed a higher frequency of coexpression in lamina III and based on similar criteria, these could belong to either Glut10 or Glut14.

Peirs et al. (2015) have described a population of deep dorsal horn cells that transiently express VGLUT3, and have shown that these are required for mechanical hypersensitivity in both neuropathic and inflammatory pain states. It will, therefore, be of interest to know whether these “transient VGLUT3” neurons overlap with the CCK cells, and also how they fit into the classification scheme of Häring et al.

### 4.3 CCK expression by PKCγ cells

Although Häring et al. (2018) found mRNA for PKCγ distributed across several different clusters (Glut2‐7), many earlier studies had identified a distinctive population of excitatory interneurons that showed PKCγ immunoreactivity (Abraira et al., 2017; Alba‐Delgado et al., 2015; Hughes, Averill, King, Molander, & Shortland, 2008; Lu et al., 2013; Malmberg et al., 1997; Martin, Liu, Wang, Malmberg, & Basbaum, 1999; Miraucourt et al., 2007; Mori, Kose, Tsujino, & Tanaka, 1990; Neumann, Braz, Skinner, Llewellyn‐Smith, & Basbaum, 2008; Polgár, Fowler, McGill, & Todd, 1999). These cells are concentrated in laminae IIi and III, and their dendrites form a dense plexus in lamina IIi. Knock‐out of PKCγ in mice leads to a reduction in mechanical and thermal hypersensitivity following peripheral nerve injury (Malmberg et al., 1997). In addition, a selective PKCγ antagonist was found to reduce the mechanical allodynia resulting from injection of strychnine into the cisterna magna in rats (Miraucourt et al., 2007). Anatomical studies have shown that PKCγ cells are innervated by myelinated low‐threshold mechanoreceptive afferents, and have provided evidence that they respond to innocuous mechanical stimuli (Neumann et al., 2008). Taken together with electrophysiological and pharmacological data (Lu et al., 2013; Miraucourt et al., 2007; Torsney & MacDermott, 2006), this has led to the suggestion that PKCγ cells form part of a polysynaptic pathway that links low‐threshold mechanoreceptive input to lamina I projection neurons. This pathway is normally closed due to feedforward inhibitory mechanisms, but these may become ineffective following nerve injury, resulting in mechanical allodynia (Lu et al., 2013). Interestingly, there is evidence that the PKCγ cells do not contribute to mechanical hyperalgesia in inflammatory pain states (Peirs et al., 2015; Peirs & Seal, 2016).

We had previously shown that many of the cells with moderate‐strong PKCγ immunoreactivity contained neurotensin (Gutierrez‐Mecinas et al., 2016). Although PPTB cells were often PKCγ‐positive, the level of PKCγ in these cells was generally much lower, and they may therefore not contribute significantly to the allodynia attributed to PKCγ, or to PKCγ‐expressing neurons. Here, we show that the great majority of cells with moderate‐high levels of PKCγ express either neurotensin or CCK. This finding was surprising, since Abraira et al. (2017) recently reported that there was minimal overlap between CCK and PKCγ cells. In their study, CCK cells were identified in a CCK^CreER^ mouse line, while PKCγ was detected with immunocytochemistry. Cre‐mediated recombination in this line is dependent on nuclear translocation of Cre following treatment with tamoxifen, and it may be that this was not sufficient in this population of CCK‐expressing cells.

Around half of the CCK+/PKCγ+ cells contained mRNA for TRH, and as this is virtually restricted to the Glut3 cluster of Häring et al. (2018), these cells presumably belong to this population. As PKCγ was expressed at significant levels by cells in their Glut2 (but not Glut1) cluster, the CCK+/PKCγ+ neurons that lacked TRH may form part of the Glut2 population. Interestingly, Alba‐Delgado et al. (2015) identified two distinct populations of PKCγ‐expressing cells in the rat spinal trigeminal nucleus, based on differences in morphological and electrophysiological properties. It will, therefore, be important to determine how the classes defined by Alba‐Delgado et al. are related to the different neuropeptide‐expressing classes identified here.

### 4.4 Role of CCK‐expressing cells in the dorsal horn

The principal function that has been attributed to CCK at the spinal level is a tonic reduction of the antinociceptive action of opioids, and this is thought to be mediated mainly by CCK_B_ receptors (Wiesenfeld‐Hallin, Xu, & Hokfelt, 2002). It has also been reported that CCK contributes to the development of tolerance to the analgesic effects of transcutaneous electrical nerve stimulation in a rat model of inflammatory arthritis, again involving an anti‐opioid mechanism (DeSantana, da Silva, & Sluka, 2010). A recent report suggests that the anti‐opioid action of CCK may involve hetero‐dimerization of μ‐opioid and CCK_B_ receptors (Yang et al., 2018). As the highest density of μ‐opioid receptors is in the superficial dorsal horn (Arvidsson et al., 1995), it is surprising that CCK‐expressing cells are far more numerous in laminae III‐IV. However, CCK is presumably released via a nonsynaptic mechanism (“volume transmission”) and may diffuse from release sites in the deeper laminae to the superficial dorsal horn.

Because the CCK cells are glutamatergic, it is likely that their main action will be mediated by synaptically released glutamate. Two recent studies have tested the effects of ablating CCK‐expressing dorsal horn neurons, in both cases using a mouse line in which Cre recombinase was knocked into the CCK locus (Abraira et al., 2017; Liu et al., 2018). Abraira et al. reported that mice in which CCK cells had been ablated could not distinguish between objects with different textures, and failed to show prepulse inhibition in response to a light air puff applied before an acoustic stimulus. Liu et al. showed that ablation of these cells reduced mechanical allodynia in the spared nerve injury model of neuropathic pain, and significantly decreased the number of dorsal horn neurons that showed Fos in response to brush stimulation in these mice. Together, these findings indicate that dorsal horn CCK cells have important roles in the perception of tactile stimuli in naïve animals, and that they are also involved in mechanical allodynia in neuropathic pain states.

The CCK cells represent a large subset of excitatory interneurons, accounting for around a third of those in deeper laminae, and it is therefore not surprising that ablation results in a dramatic behavioral phenotype. Our findings, together with those of Häring et al. (2018), suggest that CCK cells include functionally distinct subsets, such as those that co‐express PKCγ. The extensive overlap between CCK and PKCγ that we observed, taken together with earlier studies that have implicated PKCγ cells in neuropathic pain, raise the possibility that the CCK+/PKCγ+ cells may be particularly important for the development of mechanical allodynia. In future studies, it will be important to determine the contribution of the different neuropeptide‐expressing subsets of PKCγ neurons to the development of allodynia in neuropathic pain states.
